# Circulating microRNA-214 and -126 as potential biomarkers for canine neoplastic disease

**DOI:** 10.1038/s41598-017-02607-1

**Published:** 2017-05-23

**Authors:** Kazuki Heishima, Yukie Ichikawa, Kyoko Yoshida, Ryota Iwasaki, Hiroki Sakai, Takayuki Nakagawa, Yuiko Tanaka, Yuki Hoshino, Yasuhiko Okamura, Mami Murakami, Kohji Maruo, Yukihiro Akao, Takashi Mori

**Affiliations:** 1 Department of Veterinary Clinical Oncology, Faculty of Applied Biological Sciences, Gifu University, 1-1, Yanagido, Gifu, Gifu 501-1193 Japan; 2 Laboratory of Veterinary Pathology, Faculty of Applied Biological Sciences, Gifu University, 1-1, Yanagido, Gifu, Gifu 501-1193 Japan; 3 Laboratory of Veterinary Surgery, Graduate School of Agricultural and Life Sciences, The University of Tokyo, 1-1-1, Yayoi, Bunkyo-ku, Tokyo 113-8657 Japan; 4 Veterinary Teaching Hospital, Graduate School of Veterinary Medicine, Hokkaido University, Kita 18, Nishi 9, Kita-ku, Sapporo 060-0818 Japan; 5 Cooperative Department of Veterinary Medicine, Faculty of Agriculture, Iwate University, 3-18-8, Ueda, Morioka 020-8550 Japan; 6 Comparative Cancer Laboratory, Department of Animal Nursing, Faculty of Animal Nursing, Yamazaki Gakuen University, 4-7-2, Minami-osawa, Hachioji, Tokyo 192-0364 Japan; 7 United Graduate School of Drug Discovery and Medical Information Sciences, Gifu University, 1-1, Yanagido, Gifu, Gifu 501-1193 Japan

## Abstract

Circulating microRNAs in the blood may provide diagnostic and prognostic information about canine neoplastic diseases, and their profiles may be conserved between human and canine species. We performed RT-qPCR to obtain the profiles of circulating plasma microRNA-214 and -126 in total 181 cases of canine neoplastic diseases and healthy controls. MicroRNA-214 levels were high in 2 epithelial tumours (thyroid and mammary carcinomas) and 4 non-epithelial tumours (osteosarcoma, histiocytic sarcoma, chondrosarcoma, and hemangiosarcoma). In contrast, microRNA-126 levels were high in 6 epithelial tumours (mammary, hepatocellular, squamous cell, thyroid, transitional cell carcinomas, and adenocarcinoma) and 4 non-epithelial tumours (osteosarcoma, mast cell tumour, melanoma, and hemangiosarcoma). The diagnostic potential of microRNA-214 was relatively high in sarcomas, whereas that of microR-126 was high in most types of the tumours. MicroRNA-214 and -126 were prognostic predictors in 2 groups (adenocarcinoma and non-epithelial tumours except for osteosarcoma) and 3 groups (epithelial tumours, adenocarcinoma, and melanoma), respectively. Additionally, the microRNA levels did not show a strong correlation with the other clinical parameters. In conclusion, circulating microRNA-214 and -126 have the potential to be diagnostic and prognostic biomarkers for canine neoplastic diseases. Furthermore, their profiles may be key references as well for exploring novel biomarkers for human cancers.

## Introduction

Low-invasive biomarkers for obtaining diagnostic and prognostic information may have considerable importance in determining the treatment strategy for patients with neoplastic disease. Several blood-based biomarkers, such as carcinoembryonic antigen (CEA)^1^ and alpha-fetoprotein (AFP)^2^, have been identified as low-invasive biomarkers in human oncology. However, clinically available low-invasive biomarkers for canine tumours have yet to be identified. To date, some blood proteins and enzymes, such as canine C-reactive protein (CRP) and thymidine kinase, have been studied as low-invasive biomarkers for canine tumours^3^; however, these protein-based markers were found to be difficult for fully meeting the clinical demand as tumour biomarkers because their activities are easily affected by a change in the storage temperature and numerous other clinical conditions including inflammation. Identifying novel low-invasive biomarkers for canine tumours is thus of great interest in veterinary clinical oncology.

Identifying biomarkers for canine neoplastic diseases is important with respect to human oncology as well. Human and canine spontaneous tumours share many essential biological and clinical features, such as their genetics, histopathological morphology, and malignant behaviours to invade and metastasise to other organs^4^. Canine spontaneous tumours have an additional unique feature of maintaining the tumour microenvironment, including tumour heterogeneity, intact immune system, abnormal vasculature, and intratumoral hypoxic gradient, whereas conventional mouse xenograft models lack these features^4, 5^. Furthermore, the domestic dog frequently develops several tumour types that are extremely rare in humans; for instance, the incidence of sarcomas, which are malignancies derived from mesenchymal cells, is 4 times higher in dogs (17.8–20.1/100,000/year^6^) than in humans (2–5/100,000/year^7^). Given the similarities and unique features, canine spontaneous tumours have the potential to be useful models in biomarker research; and thus, developing biomarkers for canine neoplastic diseases might help toward the discovery of novel biomarkers for human neoplastic diseases.

MicroRNAs (miRNAs), a class of short-noncoding RNAs, play indispensable roles in tumorigenesis by controlling the differentiation, proliferation, and cell death of cancer cells^8^. Cancer cells not only show dysregulation of miRNAs but also release these miRNAs into the bloodstream^9^. Such “circulating miRNAs” are stabilised by their binding proteins^10^ or extracellular vesicles (EVs), such as exosomes and microvesicles (MVs)^11^; and thus, they are resistant to degradation by RNase. The levels of circulating miRNAs can accurately reflect the number of tumour cells, response to treatment, clinical stages, and tumour grades^12^. In addition, the sequences of mature miRNAs are broadly conserved among diverse species^13^. Therefore, circulating miRNAs have a great potential to be common diagnostic and prognostic biomarkers for both human and canine neoplastic diseases.

A large number of miRNAs are dysregulated in various human and canine tumours^14–16^; however, the miRNAs suitable as biomarkers to evaluate various human and canine tumours are limited to those being released into the blood and regulating fundamental mechanisms of tumour pathogenesis in both species. MicroRNA-214 (miR-214) and microRNA-126 (miR-126) regulate angiogenesis, proliferation, migration, and cell death of cancer cells; and thus, dysregulation of these 2 miRNAs critically influences tumour progression^17, 18^. Furthermore, circulating miR-214 or -126 levels are increased in 6 human tumours (multiple myeloma^19^, osteosarcoma^20^, breast^21^, gastric^22^, ovarian^23^, and non-small cell lung carcinoma^24^) and a canine sarcoma (canine hemangiosarcoma^25^). Considering the broad influence of miRNAs on cancer pathogenesis beyond histologic types and species, we hypothesized that circulating miR-214 and -126 could be potential low-invasive biomarkers common for diverse human and canine tumours.

In order to validate the versatility and practicability of miRNA-based biomarkers, it is necessary to assess the overall profiles between diverse tumour types in both humans and dogs. However, prior studies on human cancers have mainly focused on determining the levels of the miRNAs in a single or small population of specific tumours without a comparison between the tumour types^17, 18^; and few studies have attempted to obtain the miRNA profiles in a broad spectrum of tumour types. This is because humans develop limited types of cancers, and as such, it may be difficult to collect a wide variety of cancers, especially the rare ones such as sarcomas. In veterinary medicine, although dogs develop a broad range of tumours, levels of circulating miR-214 and -126 have not been explored except for our previous report on canine hemangiosarcoma^25^. We thus addressed this issue in our present study by evaluating the levels of circulating miR-214 and -126 in a wide variety of canine neoplastic diseases. The purpose of this study was to assess the potential of circulating miRNA-214 and -126 for use as diagnostic and prognostic biomarkers in various canine neoplastic diseases, as well as for obtaining referential information for exploring novel biomarkers in human cancers.

## Materials and Methods

### Homology analysis between human and canine miRNA sequences

Sequences of human and canine mature miRNAs were obtained from miRBase (version 21, http://www.mirbase.org)^26^. The sequence homology of human and canine mature miRNAs was evaluated by using Microsoft Excel (Microsoft Corporation, WA, USA).

### Sample collection

Plasma samples were primarily obtained from dogs clinically diagnosed as tumourous at the first visit to veterinary physicians at Gifu University, The University of Tokyo, Hokkaido University or Iwate University. After conducting a follow-up survey, we excluded cases without definitive diagnosis by histopathology. Histopathological examination was performed by at least 1 Japanese College of Veterinary Pathology or American College of Veterinary Pathology board-certified pathologist. Control samples were obtained from healthy dogs that presented with no specific disease. The sampling of plasma was carried out by procedures approved by the Committee of Animal Medical Centre of Gifu University. The dog owners provided their informed consent for clinical samples to be collected for this study. All experiments in this study were performed in accordance with the relevant guidelines and regulations.

### Handling of clinical samples

Blood samples were carefully collected from a jugular or cephalic vein by syringe with a 21 or 23 G needle to avoid artificial haemolysis. Whole-blood samples were added to ethylenediaminetetraacetic acid-containing tubes and gently mixed at room temperature. After the samples had been centrifuged at 3,000 rpm for 20 min at 4 °C, the plasma was carefully moved to a new 1.5-mL microfuge tube and stored at −80 °C until measurement of miRNA levels could be performed. All visually recognizable haemolysed samples were excluded from this study. To minimise the RNA degradation, we only used the samples which freeze-and-thaw was not exceeded 3 times.

### Extraction of total circulating miRNA from plasma

Total circulating RNA was extracted from plasma samples by using the NucleoSpin^®^ miRNA Plasma kit (MACHEREY-NAGEL, Düren, Deutschland) according to the standard protocol. Three hundred µL frozen plasma samples were thawed and centrifuged for 3 min at 11,000 × g to remove residual cell debris. Blood proteins in the supernatant were precipitated by using a reagent in the kit and removed by centrifugation. After adjustment of the binding conditions with isopropanol, circulating RNAs were bound to an miRNA collection column. To avoid contamination by cell-free plasma DNAs, a recombinant DNase in the kit was applied to digest these DNAs on the column. The miRNA was then eluted into 10 µL of RNase-free water. The yield of circulating miRNA from plasma was normalised by using the same amount of plasma sample (300 µL) in each extraction. The purified miRNA was directly and immediately used for the following RT process to prevent degradation of RNA before PCR step. Common methods for RNA integrity and quality check before the RT process, such as a microvolume spectrophotometer and denaturing polyacrylamide gel electrophoresis, could not be employed in the present study due to the following reasons. The yield of total RNA from plasma is usually very small, and a microvolume spectrophotometer is hardly able to detect them, unlike cellular total RNA from tissues and cells, or serum having contamination of abundant platelet-derived cellular RNAs^27^. Denaturing polyacrylamide gel electrophoresis and other methods assessing 18S and 28S ribosomal RNA are also not available to evaluate the quality of circulating miRNA because total RNA from plasma does not have such predominantly abundant RNA. cDNA was amplified immediately after the RT reaction had been finished to minimise the degradation before the PCR reaction. The RNA quality was assessed by measuring circulating miR-16 levels. miR-16 is abundantly and stably found in plasma similar to ribosomal RNA in cellular RNAs, and thus, the levels should reflect the grade of RNA degradation and thus the quality of the plasma samples.

### miRNA reverse transcription quantitative polymerase chain reaction analysis

An miRNA reverse transcription quantitative polymerase chain reaction (RT-qPCR) was performed for measuring levels of circulating miR-214, -126, and -16. For the accurate detection and quantification of these short miRNAs having only 22 nucleotides, we performed a looped-primer RT-qPCR using the TaqMan^®^ MicroRNA Assays (Applied Biosystems^®^, Thermo Fisher Scientific, MA, USA) for miR-214 (AB Assay ID 002306), miR-126 (AB Assay ID 002228) and miR-16 (AB Assay ID 000391) with the TaqMan^®^ MicroRNA Reverse Transcription Kit (Applied Biosystems^®^). miR-214, -126, and -16 were individually reverse-transcribed to cDNA by using the looped-RT primers that specifically reverse-transcribes each miRNA and add extensional sequences for the following PCR amplification. Each cDNA replicate was made in single. The RT condition was as follows. Step 1: 4 °C for 3 min; Step 2: 16 °C for 30 min; Step 3: 42 °C for 30 min; Step 4: 85 °C for 5 min followed by a 4 °C hold. The cDNA unique to each miRNA was subsequently amplified by using pre-designed forward and reverse PCR primers and TaqMan minor-groove-binder probes. These primers specifically bind to cDNA containing the sequences of each miRNA with the extension by the looped-RT primer. cDNA was diluted to 10 times at the final concentration with THUNDERBIRD^®^ Probe qPCR Mix (Cat. #QPS-101, TOYOBO, Osaka, Japan) and DNase/RNase-free distilled water. The cDNA was amplified with the measurement of fluorescence using a TaKaRa Thermal Cycler Dice^®^ Real Time System I (Takara, Shiga, Japan). The cycle conditions used in the PCR step was as follows. Step 1: 95 °C for 30 sec; Step 2: 95 °C for 5 sec; Step 3: 60 °C for 1 min; Repeat Steps 2–3 for 50 cycles. The PCR replicate was made in triplicate. The amplification efficiency of the qPCR reaction was examined in several runs and confirmed to be high enough (96–100%), whereas the efficiency was not evaluated in all runs or samples.

The threshold cycle (Ct) was automatically calculated by the second derivative maximum method to minimise the errors due to variation of a manual threshold determination and differences of background fluorescence in the samples and runs^28^. The Ct values were identified at the cycle of maximum fluorescence acceleration, the beginning point of the log-linear phase in the amplification curve.

miR-16 was selected as an internal control in the present study because it was the best internal control available for normalising plasma miRNAs according to our previous study^25^. We considered that miR-16 was a reasonable internal control for our purposes due to the following reasons. miR-16 is conserved between human and canine (Supplemental Fig. 3d). miR-16 was abundantly found in plasma^25, 29, 30^, and thus, the total amount would be stable under various biological conditions^30^. The relative quantities of miR-214 and -126 to miR-16 were determined by calculating the 2^−ΔCt^ as used for the previous analysis.

### Collection of clinical information

Clinical information including age, gender, weight, breed, histological diagnosis, haematocrit (Hct.), platelet (Plat.), fibrinogen (Fibn.), prothrombin time (PT), activated partial thromboplastin time (APTT), blood urea nitrogen (BUN), creatinine (Cre.), alanine aminotransferase (ALT), aspartate aminotransferase (AST), alkaline phosphatase (ALP), and CRP, was collected at the first visit as well as at the time of plasma sample collection. Detailed information regarding histopathological diagnosis and breeds are given in Supplemental Table 1 and Supplemental Fig. 1, respectively.

### Survival analysis

Survival information including survival time and cause of death was collected by a follow-up survey. Cases euthanized or in which death occurred by accident were excluded from the survival analysis. The Kaplan-Meier method and the log-rank test were used to assess the correlations between the levels of circulating miRNAs and survival. GraphPad Prism 7 (GraphPad Software, Inc., CA, USA) was used for the illustration of Kaplan-Meier survival curves and calculation of the P-values. The optimal cut-off values dividing the cases into the groups with high and low circulating miRNA levels were calculated by using X-tile plot software^31^ (Yale University School of Medicine, CT, USA), which assesses the optimal cut-point based on Chi-square values defined by the log-rank test. The X-tile software used in this study was version 3.6.1.

### Other statistical analysis

JMP Version 12, 64-bit (SAS Institute Inc., NC, USA) or GraphPad Prism 7 (GraphPad Software, Inc.) was used for each statistical analysis. For single comparisons, a nonparametric Mann-Whitney U test was used for comparing the medians of the 2 groups. For multiple comparison, the Kruskal-Wallis test was first used for examining whether the samples originated from the same distribution; then, Steel’s test was performed as the post-hoc test for comparing several groups with a control. The cluster analysis, dendrogram, and heatmap were illustrated by using JMP Version 12, 64-bit (SAS Institute Inc.). The clusters were determined by Ward’s method. For sets of categorical data, a chi-square test was used to examine whether the difference was significant. Receiver operating characteristic (ROC) curve analysis was used to calculate the area under the curve (AUC) value for assessing the validity of using circulating miRNAs to discriminate disease cases from healthy subjects. The Youden index was used for determining the optimal cut-off point to calculate the specificities and sensitivities in ROC curve analysis. Multivariate correlation analysis was performed to examine the correlation between miRNA levels and the other clinical parameters using JMP Version 12, 64-bit (SAS Institute Inc.). Calculated correlations were summarised to a correlation map by using Cytoscape (Version 3.4.0., Cytoscape Consortium, http://www.cytoscape.org). For further assessment of the correlation between 2 variables, a scatter plot was constructed and linear regression analysis was performed by using GraphPad Prism 7 (GraphPad Software, Inc.). The statistical methods used in this study are summarised in Supplemental Table 2. A P-value of less than 0.05 was considered to be significant.

## Results

### Mature miRNA sequences were conserved well between human and dog

First, we examined sequence homology between human and canine mature miRNAs to evaluate the similarities of miRNAs in these two species. We collected the sequences of all human and canine mature miRNAs registered in miRBase version 21 and calculated how many miRNAs conserved between human and canine. As a result, human 2588 and canine 453 mature miRNAs hit on the database, and 172 of the 453 (38.0%) canine miRNAs showed a perfect match with human homologs (Supplemental Fig. 3a). Furthermore, the canine mature miRNAs that we evaluated in the present study, miR-214, -126 and -16, showed perfectly identical sequences to human (Supplemental Fig. 3b,c,d).

### Levels of circulating miR-214 and -126 were increased in canine neoplastic disease

**Figure 1:**
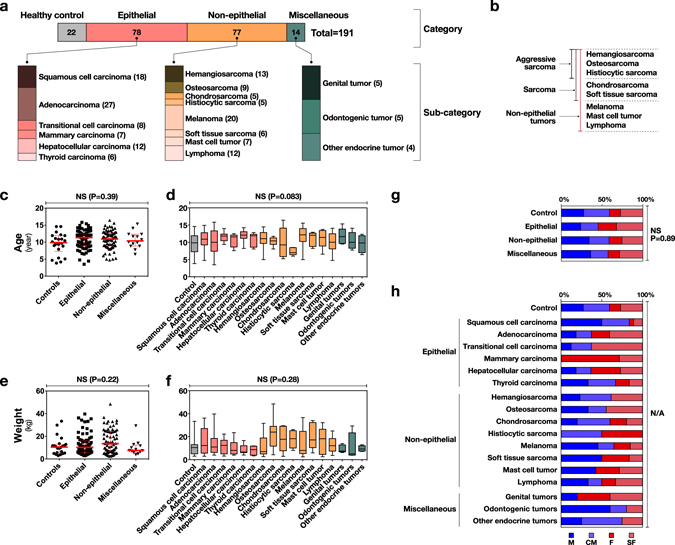
Summarised characteristics of the dogs used in this study. No obvious bias was observed with respect to age, weight, or gender of the dogs enrolled in this study. **(a)** Summary and case counts of the total 191 dogs enrolled into this study. Samples were categorised into 4 categories and 17 subcategories. **(b)** Classification used in this study for non-epithelial tumours **(c)** Swarm plot of ages (years) for the 4 categories (Kruskal-Wallis test). **(d)** Swarm plot of ages (years) between subcategories (Kruskal-Wallis test). **(e)** Swarm plot of weights (kg) between 4 categories. (Kruskal-Wallis test) **(f)** Swarm plot of weights (kg) between subcategories. (Kruskal-Wallis test) **(g)** Genders of 4 categories (Chi-square Test). **(h)** Genders of subcategories. Only mammary carcinoma cases had no male ones. Statistical analysis was not available (N/A) due to the limited number of cases in the subcategories to calculate an appropriate P-value by use of the Chi-square test. M, male; CM, castrated male; F, female; SF, spayed female.

To assess the profiles of circulating miR-214 and -126, we prospectively collected plasma samples from both tumour-bearing and healthy dogs. After definitive diagnosis by histopathology, a total of 191 samples including epithelial (n = 78), non-epithelial (n = 77), miscellaneous (n = 14) tumours, and control cases (n = 22) were evaluated (Fig. 1a and Supplemental Table 1). We further divided the non-epithelial tumours into sarcomas (hemangiosarcoma, osteosarcoma, histiocytic sarcoma, chondrosarcoma, and soft tissue sarcoma) and aggressive sarcomas (hemangiosarcoma, osteosarcoma, and histiocytic sarcoma) as illustrated in Fig. 1b. We confirmed that the samples had no obvious statistical bias regarding age (Fig. 1c,d) or weight (Fig. 1e,f). There was no statistically significant difference in gender between epithelial, non-epithelial, miscellaneous tumours, and control cases (Fig. 1g). Statistical analysis was not available to examine the difference in each subcategory due to the limited number of cases; however, the case numbers of males/castrated males were equivalent to those of females/spayed females in most subcategories. Exceptionally, most cases of squamous cell carcinoma were males/castrated males; and those of mammary carcinoma and genital tumours were females/spayed females (Fig. 1h).

**Figure 2:**
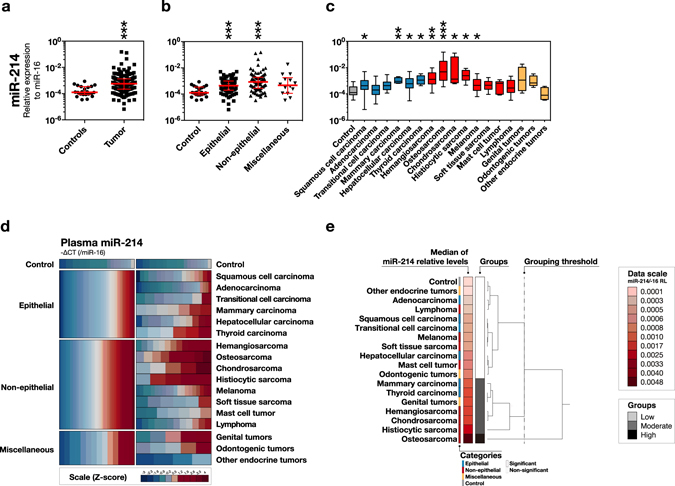
Circulating miR-214 profiles for canine tumours. Circulating miR-214 levels (−∆CT, relative to circulating miR-16 levels) were mainly increased in non-epithelial tumours, especially the sarcomas. **(a)** Swarm plot of circulating miR-214 levels comparing tumour and control. ***P < 0.001 (Mann-Whitney U test) **(b)** Swarm plot of circulating miR-214 levels comparing the groups of epithelial, non-epithelial, miscellaneous tumours, and controls. *P < 0.05, ***P < 0.001 (Steel’s Test) **(c)** Box plot of circulating miR-214 levels comparing the subcategories. *P < 0.05, **P < 0.01, ***P < 0.001 (Steel’s Test). **(d)** Heatmap overview of circulating miR-214 levels. **(e)** Hierarchical cluster analysis of circulating miR-214 levels. The representative values used were the medians of miR-214 levels. The dendrogram was illustrated by Ward’s method, and categorised into 3 groups based on the indicated threshold.

We next performed miRNA RT-qPCR to determine the profiles of circulating miR-214 and -126 in the tumour-bearing and control cases. We confirmed that miR-16, the internal control, was stably detected in these samples (Supplemental Fig. 4) and found that miR-214 levels in the tumour-bearing cases were significantly higher than those in the control cases (Fig. 2a,d). miR-214 was significantly increased in both epithelial and non-epithelial tumours, and the non-epithelial tumours showed the highest median miR-214 levels (Fig. 2b,d). In the subcategories, 4 types of non-epithelial tumours (osteosarcoma, histiocytic sarcoma, chondrosarcoma, and hemangiosarcoma) and 2 kinds of epithelial tumours (thyroid carcinoma and mammary carcinoma) showed a high or moderate increase in miR-214 levels with statistical significance (Fig. 2c,e). Genital tumours were classified into the moderately increased group; however, the difference was not significant. Hepatocellular carcinoma, melanoma, and squamous cell carcinoma were grouped as low-level, although their medians were significantly different from those of the controls.

**Figure 3:**
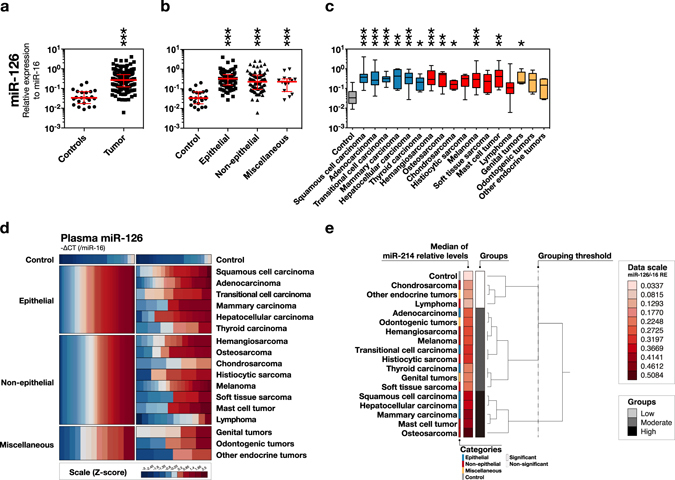
Profile of circulating miR-126 for canine tumours. Circulating miR-126 levels (-∆CT, relative to circulating miR-16 levels) were high in most of the tumour types. **(a)** Swarm plot of circulating miR-126 levels comparing tumour and controls. ***P < 0.001 (Mann-Whitney U test) **(b)** Swarm plot of circulating miR-126 levels comparing the groups of epithelial, non-epithelial, miscellaneous tumours, and controls. ***P < 0.001 (Steel’s Test) **(c)** Box plot of circulating miR-126 levels comparing the subcategories. *P < 0.05, **P < 0.01, ***P < 0.001 (Steel’s Test). **(d)** Heatmap overview of circulating miR-126 levels. **(e)** Hierarchical cluster analysis of circulating miR-126 levels. The representative values used were the medians of miR-126 levels. The dendrogram was illustrated by Ward’s method, and categorised into 3 groups based on the indicated threshold.

As observed for circulating miR-214, circulating miR-126 levels were significantly high in tumour-bearing cases (Fig. 3a,d). Circulating miR-126 was generally increased in most histological subtypes, and the epithelial tumours showed the highest median circulating miR-126 levels (Fig. 3b,d). Six subtypes of epithelial tumours (mammary, hepatocellular, squamous cell, thyroid, transitional cell carcinoma, and adenocarcinoma) and 4 subtypes of non-epithelial tumours (osteosarcoma, mast cell tumour, melanoma, and hemangiosarcoma) showed a statistically high or moderate increase in miR-126 levels (Fig. 3c,e). Histiocytic sarcoma, odontogenic tumours, and soft tissue sarcoma were classified into the moderately increased groups; however, their medians were not significantly different from those of the controls. Chondrosarcoma was classified as low-level, whilst the median was significantly different from that of the controls.

### Difference in circulating miR-214 and -126 profiles between epithelial and non-epithelial tumours

**Figure 4:**
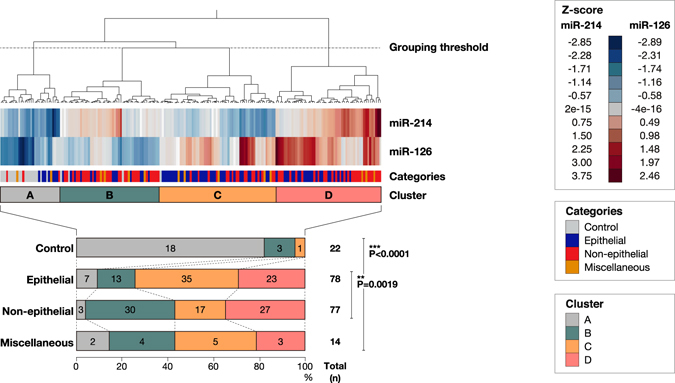
Heatmap and hierarchical cluster analysis of circulating miR-214 and -126 levels. Levels of circulating miR-214 and -126, showing different profiles between epithelial and non-epithelial groups. Heatmap and dendrogram were illustrated by Ward’s method using JMP software version 12.3. Samples were classified into 4 clusters based on the indicated threshold line. Relative levels (−∆CT, relative to circulating miR-16) were normalised by the Z-score. Statistical significance between each category was calculated by the Chi-square test (**P < 0.01, ***P < 0.001).

We next classified the cases into 4 clusters (A, B, C, and D) based on hierarchical cluster analysis using both circulating miR-214 and -126 levels. The result showed that the groups of epithelial, non-epithelial, miscellaneous tumours, and controls showed significantly different profiles (P < 0.0001, Fig. 4). The majority of controls (81.8%, 18 out of 22 cases) were classified into group A. The majority of epithelial tumour cases (74.4%, 58 out of 78 cases) and non-epithelial tumours (74%, 57 out of 77 cases) were classified into group C-D and group B-D, respectively (P = 0.0019). The miscellaneous tumour cases were distributed to each cluster in a similar ratio to that observed in the total cases.

### Diagnostic potential of circulating miR-214 and -126 in canine neoplastic disease

**Figure 5:**
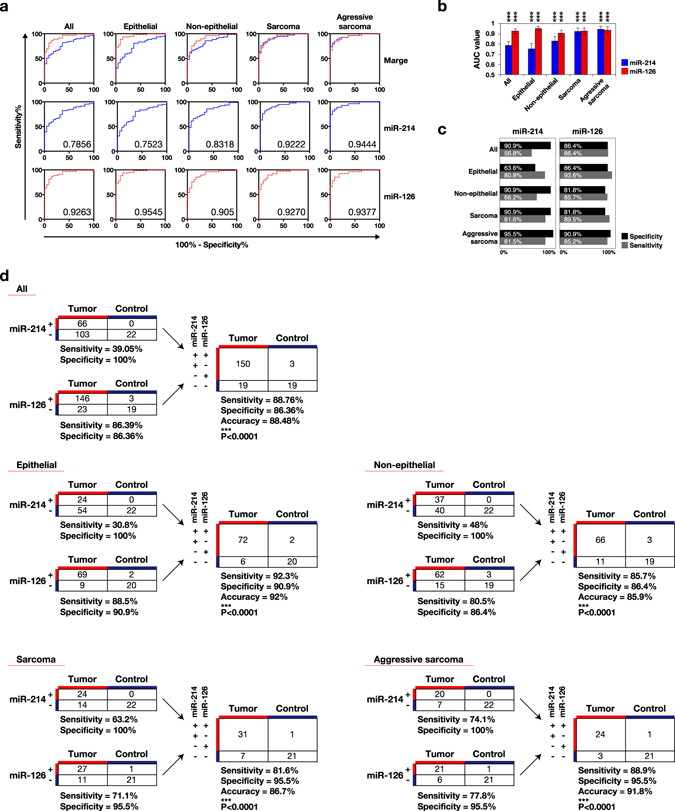
Diagnostic accuracies of circulating miR-214 and -126. Circulating miR-214 and -126 discriminated tumour from control cases with high accuracies. **(a)** ROC curve analysis of circulating miR-214 and -126 in the case of all types of tumours: epithelial, non-epithelial tumours, sarcomas (hemangiosarcoma, osteosarcoma, chondrosarcoma, histiocytic sarcoma, and soft tissue sarcoma), and aggressive sarcomas (hemangiosarcoma, osteosarcoma, histiocytic sarcoma). Blue lines and red dotted lines indicate the results of circulating miR-214 and miR-126, respectively. The numbers shown under the lines indicate the AUC value of each data set. **(b)** Summary of the AUC values from ROC curve analysis. Error bars indicate the standard error of each mean. P-values were calculated by using an unpaired two-tailed student’s T-test (***P < 0.0001). The null hypothesis was that the calculated AUC value was 0.5. **(c)** Specificities and sensitivities of circulating miR-214 and -126 at the optimal cut-offs. Each optimal cut-off was determined by using the Youden index. **(d)** The combined use of circulating miR-214 and -126 improved the diagnostic accuracies and sensitivities in each group. P-values were calculated by performing a chi-square test (***P < 0.0001).

Next, we assessed the diagnostic potential of circulating miR-214 and -126. ROC curve analysis showed that the AUC values of circulating miR-214 in discriminating tumour from control cases was high in the non-epithelial tumours (AUC = 0.83), especially in the sarcomas (hemangiosarcoma, osteosarcoma, chondrosarcoma, histiocytic sarcoma, and soft tissue sarcoma; AUC = 0.92) and aggressive sarcomas (hemangiosarcoma, osteosarcoma, and histiocytic sarcoma; AUC = 0.94), but relatively low in the epithelial tumours (AUC = 0.75) and the group including all types of tumours (AUC = 0.79, Fig. 5a,b). Circulating miR-214 showed high specificities (90.9–95.5%) and sensitivities (81.5–81.6%) in the sarcomas and aggressive sarcomas, but it showed relatively low specificities (63.6–90.9%) and sensitivities (56.8–80.8%) in the epithelial tumours and the group including all types of tumours (Fig. 5c). The AUC values of circulating miR-126 in discriminating tumour from control cases were generally high throughout all types of the tumours (AUC = 0.93), with the epithelial tumours showing the highest AUC value, 0.95 (Fig. 5a,b). Circulating miR-126 generally had high specificities (81.8–90.0%) and sensitivities (85.2–93.6%) at the optimal cut-off point (Fig. 5c). Combining both miRNAs further increased the accuracies and sensitivities: the accuracy, sensitivity, and specificity increased to 88.48%, 88.76%, and 86.36% in the group including all tumour types, 92%, 92.3%, and 90.9% in the epithelial tumours, 85.9%, 85.7%, and 86.4% in the non-epithelial tumours, 86.7%, 81.6%, and 95.5% in the sarcomas, and 91.8%, 88.9%, and 95.5% in the aggressive sarcomas (Fig. 5d).

### Prognostic potential of circulating miR-214 and -126 in canine neoplastic disease

**Figure 6:**
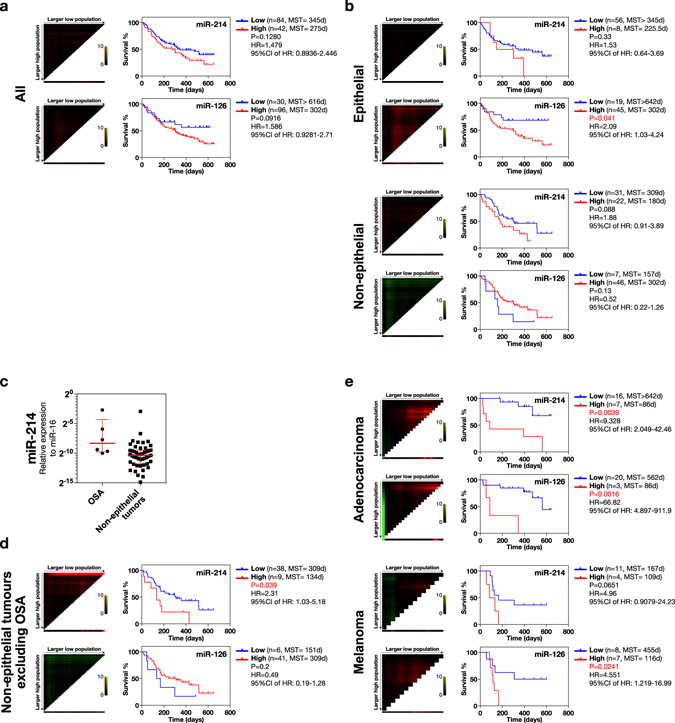
Survival analysis using Kaplan-Meier survival curve and X-tile plot. The cases with high levels of circulating miR-214 showed significantly shorter survival time in the cases of adenocarcinoma and the non-epithelial tumours without osteosarcoma. The cases with high levels of circulating miR-126 showed significantly shorter survival times in the cases of epithelial tumours, adenocarcinoma, and melanoma. **(a)** Survival times between the cases with high- and low-circulating miR-214 and -126 in the group including all types of tumours. **(b)** Survival times between the cases with high- and low-circulating miR-214 and -126 levels for the epithelial and the non-epithelial tumours. **(c)** Comparison of the levels of circulating miR-214 between osteosarcomas (OSA) and the other non-epithelial tumours. **(d)** Survival times between the cases with high- and low-circulating miR-214 and -126 levels for the non-epithelial tumours excluding osteosarcomas. **(e)** Survival times between the cases with high- and low-circulating miR-214 and -126 levels for adenocarcinomas and melanomas. All MST values (days) were calculated from the analysis using Kaplan-Meier survival curves. The optimal cut-offs to determine the groups of high- and low-miRNA levels were calculated by use of the X-tile plot. The hazard ratios and P-values were calculated by using the Log-rank test.

We assessed the potential of circulating miR-214 and -126 as prognostic biomarkers for canine neoplastic disease. First, we compared the survival duration between the cases with high- and low-circulating miR-214 or −126 in the group including all types of tumours; however, the duration of survival was not significantly different between these 2 groups (Fig. 6a). On the other hand, the epithelial tumour cases with high circulating miR-126 had a significantly shorter survival duration (P = 0.041) than those with low miR-126 (Fig. 6b). In the non-epithelial tumour cases, the survival duration between high and low groups was not significantly different (Fig. 6b); however, we noticed that the osteosarcoma cases had extraordinarily high levels of circulating miR-214 compared to the other non-epithelial tumours (Fig. 6c). Thus, we speculated that the high levels of circulating miR-214 in osteosarcoma cases might have obscured the survival difference in the case of other non-epithelial tumours. Therefore, we re-analysed the survival differences in non-epithelial tumours except for osteosarcoma cases. As a result, the non-epithelial tumour cases with a high level of circulating miR-214 showed a significantly shorter survival duration when the osteosarcoma cases were excluded (Fig. 6d). We were able to analyse the survival durations of 2 histological subtypes, i.e., adenocarcinoma and melanoma, because these subtypes occurred in a relatively large number of cases compared to the other subtypes. Adenocarcinoma cases with high miR-214 and -126 levels survived for a significantly shorter time (Fig. 6e). As to melanoma cases, adenocarcinoma cases with high miR-126 levels had a significantly shorter survival duration, whilst those cases with high miR-214 levels did not (Fig. 6e). We also determined the levels of those miRNAs in each clinical stage of melanoma for reference purpose, although the statistical analysis could not be made due to the limited number of cases at each clinical stage (Supplemental Fig. 2). Taken together, these results showed that the cases with high miR-214 levels had a shorter survival duration in the group of non-epithelial tumours excluding osteosarcomas and in the adenocarcinomas of the epithelial group; furthermore, the adenocarcinoma and melanoma cases with high miR-126 levels had a shorter survival time than those with low levels.

### Levels of circulating miR-214 and -126 did not show a strong correlation with other clinical parameters

**Figure 7:**
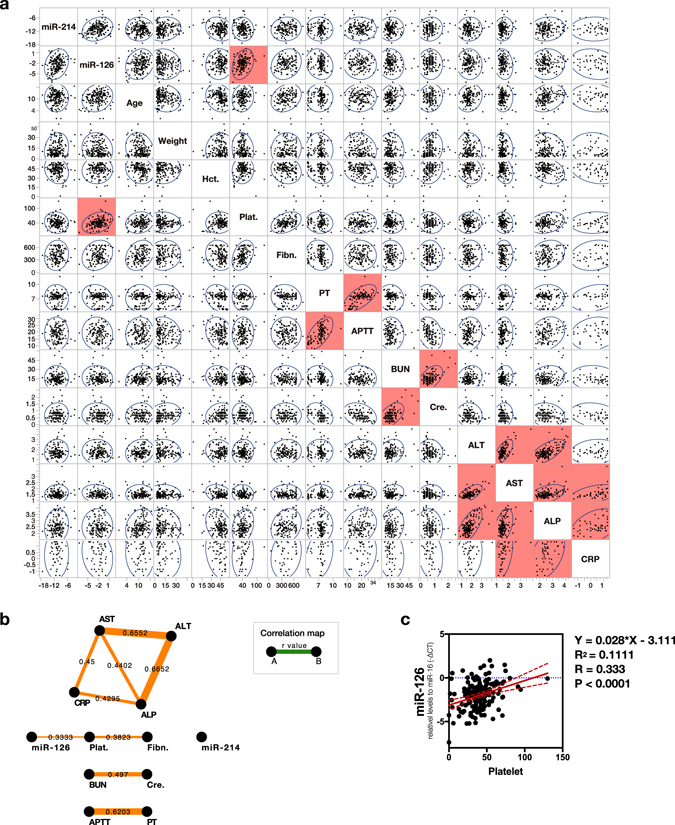
Correlation analysis between the circulating miR-214, -126 levels, and various clinical parameters. Circulating miR-214 and -126 levels did not show a strong correlation with the other clinical parameters. **(a)** Multivariate correlation analysis using scatter plots for circulating miR-214, -126 levels, age, weight, Hct., Plat., Fibn., PT, APTT, BUN, Cre., ALT, AST, ALP, and CRP. Blue circles indicate trends of scatter points. Areas given in red indicate that the 2 parameters show a significant correlation (P < 0.05, Pearson product-moment correlation coefficient). **(b)** Map for correlation between the parameters. The correlations between parameters connected to each other with bands are indicated. R-values are displayed in the centre between 2 parameters, and band thickness represents the degree of correlation. R-values only with statistical significance are illustrated on the map. **(c)** Linear regression analysis for platelet counts and circulating miR-126 levels. P-value was calculated based on the null hypothesis that the slope of the regression curve is 0.

There would be a potential weakness of circulating miRNAs as tumour biomarkers if their levels were to be affected by various other clinical parameters such as age, weight, and concurrent disorders such as inflammation or hepatic or renal damage. We thus assessed the correlation between the levels of circulating miRNAs and various parameters including age, weight, and the values from blood chemistry analysis, such as erythrocyte volume (Hct.), coagulation (Plat., PT, and APTT), kidney function (BUN and Cre.), hepatic injury (ALP, ALT, and AST), and inflammation (CRP). The results showed that the levels of circulating miR-214 and -126 were not strongly correlated with most of the clinical parameters evaluated (Fig. 7a,b). Only platelet counts showed significant correlation with the levels of circulating miR-126 (P < 0.0001); however, linear regression analysis showed that the platelet counts had a relatively small impact on determining miR-126 levels, as the coefficient of determination was low (R^2^ = 0.1111) indicating that the regression curve explained only 11% of total samples (Fig. 7c).

## Discussion

Here, we revealed the profiles of circulating miR-214 and -126 in dogs with tumours of 17 histopathological subtypes and demonstrated that both miRNAs showed the potential of being biomarkers for canine neoplastic diseases. The levels of circulating miR-214 were increased in non-epithelial tumours, especially sarcomas. In contrast, the levels of circulating miR-126 were increased in a wide variety of canine tumours. Furthermore, circulating miR-214 and -126 showed high diagnostic accuracies in sarcomas (specificities, 90.9–95.5%; sensitivities, 81.5–81.6%) and all types of canine tumours (specificity, 86.4%; sensitivity, 86.4%), respectively. These specificities and sensitivities were relatively high compared to several biomarkers established in human medicine, such as CEA for colorectal cancer^32^ (specificity, 87%; sensitivity, 36%) and pancreatic carcinoma^33^ (specificities, 66.4–87.3%; sensitivities, 48.4–71.0%), the combination of CEA, NSE, CYFRA21-1, and CA-125 for lung cancer^34^ (specificity, 83.9%; sensitivity, 34.8%), and AFP for hepatocellular carcinoma^35^ (specificity, 80–94%; sensitivity, 41–65%). In addition, circulating miR-214 and -126 were prognostic predictors in 2 groups (adenocarcinoma and non-epithelial tumours except for osteosarcoma) and 3 groups (adenocarcinoma, other epithelial tumours, and melanoma), respectively. These 2 miRNAs did not show a strong correlation with the other clinical parameters. Given that many human and canine miRNAs share identical sequences, the profiles that we revealed here provide foundational information for developing novel miRNA biomarkers for both human and canine tumours; the future addition of other circulating miRNAs to these miRNAs has the potential to improve diagnostic and prognostic accuracy.

Circulating miR-214 has been suggested to be a diagnostic^21, 22^ and prognostic^19, 20^ biomarker for various human cancers. In this study, we showed that levels of circulating miR-214 increased, and showed high diagnostic accuracies, in the dogs with non-epithelial tumours, especially sarcomas such as hemangiosarcoma, osteosarcoma, chondrosarcoma, and histiocytic sarcoma. The cases of epithelial tumours, such as mammary and thyroid carcinomas, also showed increased levels of circulating miR-214. However, other epithelial cancers showed relatively low levels of circulating miR-214. These results suggest that circulating miR-214 would be a potentially useful diagnostic biomarker for canine sarcomas, whilst it would not be suitable for use as a screening test or diagnostics for the other types of tumour. Survival analysis showed that the adenocarcinoma cases and cases of non-epithelial tumours (excluding osteosarcoma) with a high level of circulating miR-214 had a short survival time, suggesting that circulating miR-214 would have prognostic value for these canine tumours. This result is consistent with previous reports indicating that miR-214 contributes to tumorigenesis by regulating apoptosis^15, 36^, proliferation^37^, and migration^37^, as well as having an impact on prognosis^19, 20^ in diverse human cancers. Canine osteosarcoma showed outstandingly high levels of circulating miR-214 compared to the other non-epithelial tumours; moreover, the non-epithelial tumours showed a significant survival difference only when the osteosarcoma population was excluded, suggesting that osteosarcoma has distinct profiles of circulating miR-214 from the other non-epithelial tumours. It was reported that miR-214 regulates osteoblastic differentiation of mesenchymal stem cells^38^, and the level of circulating miR-214 is increased in a genetically engineered mouse model of osteosarcoma and in human osteosarcoma patients^20^. Accordingly, the high levels of circulating miR-214 in dogs with osteosarcoma may suggest a firm association between miR-214 and tumorigenesis in canine osteosarcomas. Adenocarcinoma cases with high levels of circulating miR-214 had a significantly shorter survival time, although only a few cases of them classified into the group with a high or moderate increase of circulating miR-214 in the cluster analysis. This result suggests that miR-214 estimates the prognosis but is not suitable for the diagnosis of canine adenocarcinoma. Given that exosomal miR-214 induces angiogenesis^39^, abundant extracellular miR-214 may contribute to the local invasion and metastasis through accelerating tumour angiogenesis in canine adenocarcinomas.

Circulating miR-126, as well as miR-214, has been suggested to be a diagnostic biomarker for human neoplastic disease^24^. In this study, the levels of circulating miR-126 in various canine tumours increased and showed high diagnostic accuracies, although chondrosarcoma and lymphoma showed relatively low levels of this miRNA. The results suggest that circulating miR-126 could be a diagnostic and screening biomarker for a broad range of canine tumours except for chondrosarcoma and lymphoma. The high levels of circulating miR-126 associated with the short survival time of the dogs with adenocarcinoma, other epithelial tumours or melanoma, suggest that the level of circulating miR-126 could serve to estimate the prognosis for dogs with these tumours. Given that miR-126 regulates apoptosis^40^, cell migration^41^, and proliferation^42^ in various human cancers, the results suggest that miR-126 might also contribute to tumorigenesis in these canine tumours.

Circulating miR-214 and -126 showed different profiles between the epithelial and non-epithelial tumours in this study: the level of circulating miR-214 increased mainly in the sarcomas; in contrast, circulating miR-126 increased in most types of the tumours examined. These results suggest the further application of circulating miR-214 and -126 to determination of the origin of primary tumours, although more detailed information on the profiles will be necessary for such clinical application.

Soluble (Non-EV-carried) miRNAs, unlike exosomal or MV-carried miRNAs, were once suggested to be unsuitable as biomarkers, because of their potential fragility and the unspecified influence of concurrent disease. However, more recent papers have indicated that soluble miRNAs are stabilised by binding proteins such as AGO1 and AGO2, and thus can be potentially stable biomarkers as well^43^. In the current study, we measured the total circulating plasma miRNAs, which included both EV-carried and soluble miRNAs, and demonstrated that they also had the potential to be accurate biomarkers. Moreover, multivariate correlation analysis showed that the levels of circulating miR-214 and -126 showed no correlation with most of the clinical parameters investigated in this study. These results suggest that the levels of circulating miR-214 and -126 were independent of common concurrent diseases associated with neoplasia, such as anaemia, inflammation, and hepatic and renal disorders; thus, they might be able to accurately assess the state of the tumour without noise from concurrent disease. Only the platelet count showed a statistically significant correlation with the levels of circulating miR-126 (P < 0.0001); however, the platelet counts had a relatively small impact on determining the miR-126 levels (R^2^ = 0.1111). The results reflected a previous finding that platelets contain miR-126 in their cytoplasm^44^, and suggest that miR-126 could leak out into the plasma from the platelets. Therefore, the counts and disorders of platelets possibly affected the levels of circulating miR-126, although their impact might be negligibly small. Taken together, the results suggest that circulating miR-214 and -126, which include both EV-carried and non-EV-carried forms, would be biomarkers suitable for evaluation of the tumour state with minimal effects from any concurrent disease.

Canine tumours have been advocated as an ideal model for human tumours in that they have biological and clinical behaviours, as well as a tumour microenvironment, similar to those for human tumours^4, 5^. In the present study, the canine profiles of miR-214 and -126 were consistent with several previous reports on human cancers. For instance, circulating miR-214 was increased in canine mammary carcinomas and osteosarcomas; and these results agree with previous reports showing that miR-214 levels are increased in patients with breast cancer^21^ and osteosarcoma^20^. Also, the level of circulating miR-126 was increased in canine melanomas, which corresponds with the report that circulating miR-126 is also increased in human metastatic sporadic melanoma^45^. Furthermore, there are also other reports indicating that circulating miR-214 is increased in human gastric cancers^22^, ovarian cancers^23^, and myeloma^19^, and that circulating miR-126 also is increased in human non-small cell lung carcinoma^24^. However, we were not able to assess the similarities between these human tumour types and their canine homologs due to the small number of the latter included in this study (Supplemental Table 3)^19, 21–24, 45–51^. These findings lead us to conjecture that canine and human tumours share not only their clinical and histologic features but also the profiles of circulating miRNAs, although further validation is necessary. The canine profiles of neoplastic disease thus might serve as useful references for finding biomarkers in human tumours including sarcomas.

We acknowledge several limitations in this study. First, although we collected a total of 191 cases in this study, some subcategories included a relatively small number of cases; thus, the results might not represent the actual clinical utilities in such subcategories. However, our results provide and overall perspective of the circulating miR-214 and -126 profiles in canine neoplastic disease, and would help in an additional large-scale study of each tumour type to determine the true clinical usage of the miRNAs in veterinary clinical oncology. Second, this study included only healthy controls but not any non-neoplastic disease controls, such as inflammatory, infectious, and endocrine disease. Although we demonstrated that the other established clinical parameters did not show strong correlation with the levels of circulating miR-214 and -126 in this study, it is still possible that the levels of these circulating miRNAs might increase or decrease in other non-neoplastic diseases: several studies examining human diseases reported that the level of circulating miR-214 is decreased in coronary artery disease^52^, as well as that circulating miR-126 is increased in moyamoya disease^46^, allergic rhinitis, and asthma^50^ but decreased in atherosclerosis^47^, atrial fibrillation, heart failure^51^, and type-2 diabetes mellitus^48, 49^. Future studies interrogating these miRNA profiles in non-neoplastic diseases will provide for the practical usage of these circulating miRNAs as cancer biomarkers. Third, it is still unclear whether these miRNAs are released from the tumour cells themselves. Our previous study in canine hemangiosarcoma suggested that circulating miR-214 and -126 are released from the tumour cells themselves: hemangiosarcoma cell lines released miR-214 and -126 into the surrounding environment, and surgical resection of the primary tumour decreased levels of these circulating miRNAs in the dogs with hemangiosarcoma^25^. In this present study, however, we evaluated only the levels of circulating miRNAs, not the levels in the primary tumour or in cell lines of each tumour type. Thus, in the other tumour types except for hemangiosarcoma, these miRNAs may be released from certain components of the tumour tissue, such as tumour-associated endothelial cells, inflammatory cells or fibroblasts, or by some condition associated with the neoplastic disease. The miRNAs, however, still have diagnostic or prognostic potential as tumour biomarkers whether they are released from the tumour cells themselves or not. Addressing the direct aetiology of the increase in these miRNA levels would accelerate our understanding of these miRNAs and their clinical usage. Finally, although miR-214 and -126 levels normalised by miR-16 showed a significant utility in canine cancer diagnosis and prognosis, there might be better internal controls other than miR-16 for these circulating miRNAs. Currently, the information regarding internal controls for normalising canine plasma miRNAs is very limited; however, further validation regarding the internal control might improve the diagnostic and prognostic potential of these miRNAs.

In conclusion, we have shown that circulating miR-214 and -126 have the potential to be diagnostic and prognostic biomarkers for canine neoplastic disease. Their profiles may also prompt future exploration for novel biomarkers for human cancer.

## Electronic supplementary material

Supplemental materials
