# Supplementary material for: Circulating microRNA-214 and -126 as potential biomarkers for canine neoplastic disease
Source: Sci Rep. 2017 May 23;7:2301. doi: 10.1038/s41598-017-02607-1 (PMC5442106; doi:10.1038/s41598-017-02607-1)
Supplement: Supplementary file 1 — Supplemental materials [file 41598_2017_2607_MOESM1_ESM.pdf]

Supplementary Materials

Circulating microRNA-214 and -126 as potential biomarkers for canine neoplastic disease

Kazuki Heishima,[1,*§] Yukie Ichikawa,[1,§] Kyoko Yoshida,[1] Ryota Iwasaki,[1] Hiroki Sakai,[2] Takayuki Nakagawa,[3] Yuiko Tanaka,[3] Yuki Hoshino,[4] Yasuhiko Okamura,[5] Mami Murakami,[1] Kohji Maruo,[1,6] Yukihiro Akao,[7] and Takashi Mori[1]

[1] Department of Veterinary Clinical Oncology, Faculty of Applied Biological Sciences, Gifu University, 1-1, Yanagido, Gifu, Gifu 501-1193, Japan
[2] Laboratory of Veterinary Pathology, Faculty of Applied Biological Sciences, Gifu University, 1-1, Yanagido, Gifu, Gifu 501-1193, Japan
[3] Laboratory of Veterinary Surgery, Graduate School of Agricultural and Life Sciences, The University of Tokyo, 1-1-1, Yayoi, Bunkyo-ku, Tokyo 113-8657, Japan
[4] Veterinary Teaching Hospital, Graduate School of Veterinary Medicine, Hokkaido University, Kita 18, Nishi 9, Kita-ku, Sapporo 060-0818, Japan.
[5] Cooperative Department of Veterinary Medicine, Faculty of Agriculture, Iwate University, 3-18-8, Ueda, Morioka 020-8550, Japan
[6] Comparative Cancer Laboratory, Department of Animal Nursing, Faculty of Animal Nursing, Yamazaki Gakuen University, 4-7-2, Minami-osawa, Hachioji, Tokyo 192-0364, Japan
[7] United Graduate School of Drug Discovery and Medical Information Sciences, Gifu University, 1-1, Yanagido, Gifu, Gifu 501-1193, Japan

[*] Corresponding author.
[§] These authors equally contributed to this work.

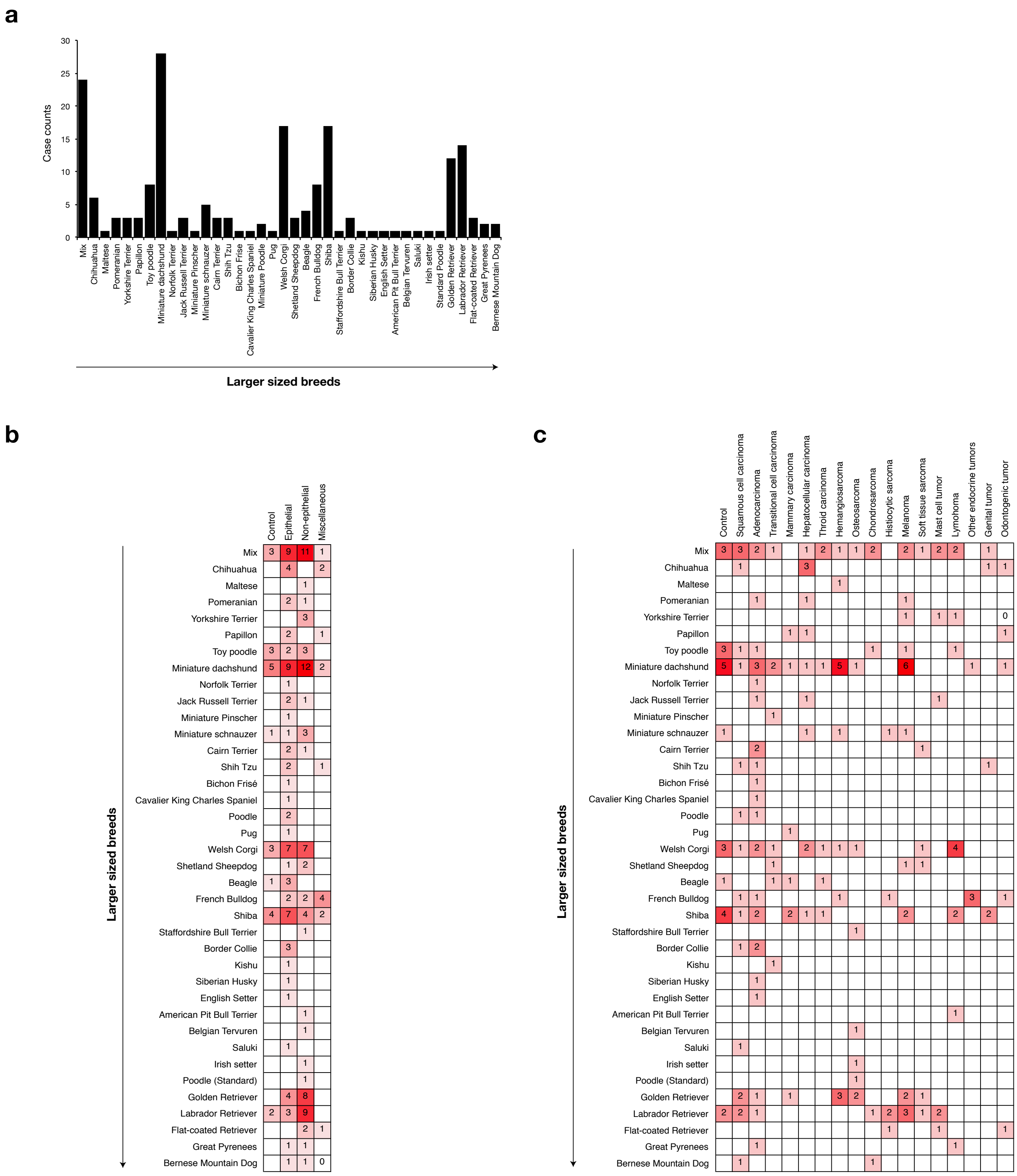

Supplemental Figure 1. Breeds of dogs enrolled in this study

Various breeds were included in this study. The top 6 major breeds were Miniature Dachshund, Mixed-breed, Welsh Corgi, Shiba, Labrador, and Golden Retriever. The breeds were arranged in order of ascending average body weight. (a) Total case number of each breed for each tumour type is given. (b) Distribution and case number of breeds in each category. (c) Distribution and case number of breeds in each subcategory.

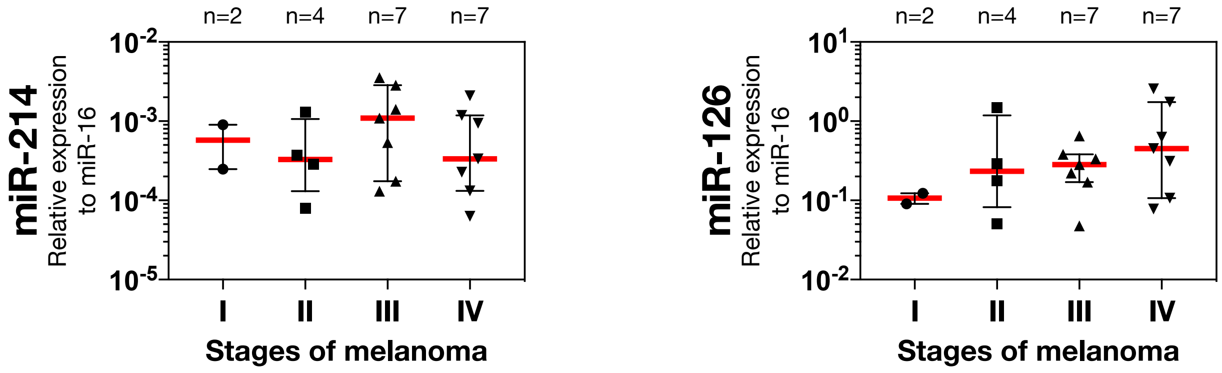

Supplemental Figure 2. Levels of circulating miR-214 and -126 in each clinical stage of melanoma

Statistical analysis was not available due to the small number of cases included in stage 1. The median levels of circulating miR-126 calculated in this study increased stage-dependently but the levels of circulating miR-214 did not. The red centre bars and error bars indicate the median and interquartile range, respectively. The staging was based on the World Health Organization staging scheme for dogs.

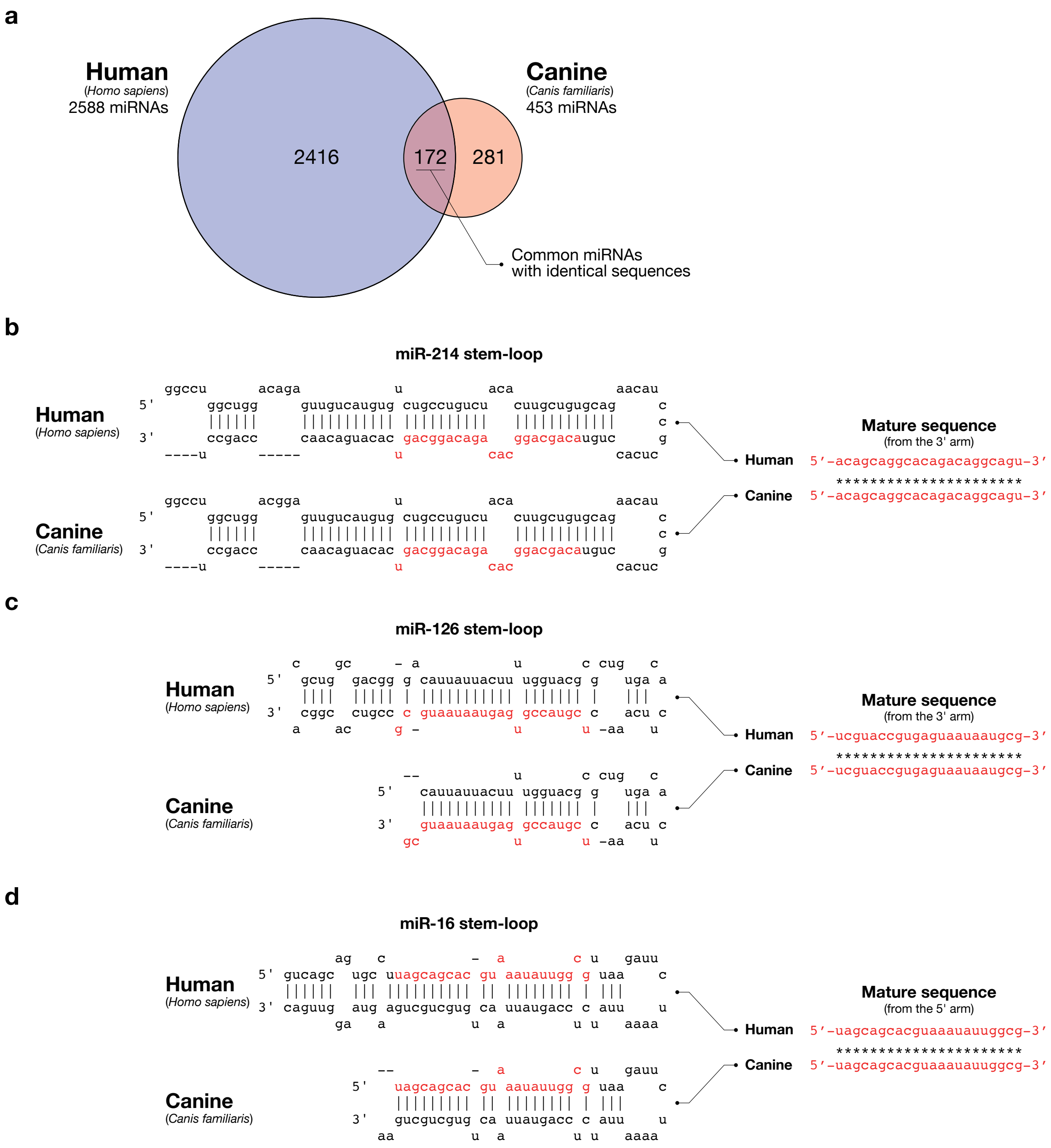

Supplemental Figure 3. Homology between human and canine miRNA sequences

Mature miRNA sequences were conserved well between human and canine. Canine mature miR-214, -126, and -16 showed perfectly identical sequences to human ones, whereas the stem-loop sequences of these miRNAs were slightly different between human and canine. (a) Venn diagram comparing human 2588 and canine 453 mature miRNA sequences registered in the database of miRBase version 21. Canine miRNAs showed a perfect match with human ones in 172 of the total 453 miRNAs (38.0%). The blue and red circle represent the groups of human and canine miRNAs, respectively. (b) Human and canine miR-214 stem-loops and the mature sequences from 3' arm. (c) Human and canine miR-126 stem-loops and the mature sequences from 3' arm. (d) Human and canine miR-16 stem-loops and the mature sequences from 5' arm. The red letters in the stem-loops indicate the mature sequences in each miRNA.

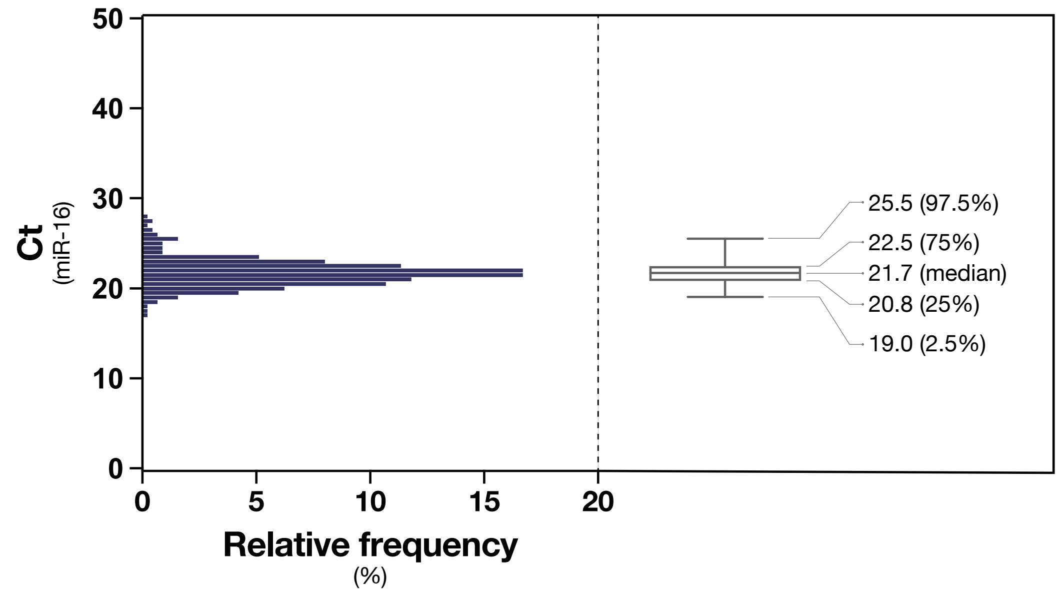

Supplemental Figure 4. Ct value distribution of circulating miR-16

Histogram and Boxplot showing a Ct value variability of circulating miR-16, the internal control in the present study. Circulating miR-16 was stably detected in the plasma samples from the dogs bearing cancers. The median Ct value was 21.7, and the Ct values ranged 20.8–22.5 (Difference: 1.7) in half of the cases. The 2.5–97.5 percentile was 19.0–25.5 (Difference: 6.5).

Categories (n)	Subcategories (n)	Diagnosis (n)	Primary site (n)
Control (22)	-	-	-
Epithelial (78)	Squamous cell carcinoma (18)	Squamous cell carcinoma (18)	Oral cavity (5)<br>Nasal cavity (4)<br>Skin (3)<br>Tonsil (3)<br>Anus (2)<br>Tongue (1)
	Adenocarcinoma (27)	Adenocarcinoma (27)	Anal sac (7)<br>Nasal cavity (5)<br>Rectum (5)<br>Gastric (3)<br>Pancreas (2)<br>Salivary gland (2)<br>Lung (1)<br>Prostate gland (1)<br>Skin (1)
	Transitional cell carcinoma (8)	Transitional cell carcinoma (8)	Bladder (5)<br>Prostate gland (2)<br>Kidney (1)
	Mammary carcinoma (7)	Mammary carcinoma (7)	Mammary gland (7)
	Hepatocellular carcinoma (12)	Hepatocellular carcinoma (12)	Liver (12)
	Throid carcinoma (6)	Thyroid follicular cell carcinoma (6)	Thyroid gland (6)
Non-epithelial (77)	Hemangiosarcoma (13)	Hemangiosarcoma (13)	Spleen (10)<br>Abdominal cavity (1)<br>Kidney (1)<br>Subcutis (1)
	Osteosarcoma (9)	Osteosarcoma (9)	Extremities (5)<br>Cranial bone (2)<br>Nasal cavity (2)
	Chondrosarcroma (5)	Chondrosarcroma (5)	Nasal cavity (2)<br>Oral cavity (2)<br>Extremities (1)
	Histiocytic sarcoma (5)	Histiocytic sarcoma (5)	Bone (2)<br>Joint (2)<br>Thoracic cavity (1)
	Melanoma (20)	Malignant melanoma (17)<br>Amelanotic malignant melanoma (3)	Oral cavity (16)<br>Tongue (2)<br>Lip (1)<br>Nailbed (1)
	Soft tissue sarcoma (6)	Hemangiopericytoma (5)<br>Fibrosarcoma (1)	Subcutis (5)<br>Spleen (1)
	Mast cell tumor (7)	Mast cell tumor (7)	Skin (6)<br>Lip (1)
	Lymphoma (12)	B-cell lymphoma (5)<br>T-cell lymphoma (7)	Lymph node (9)<br>Colon (1)<br>Skin (1)<br>Uterus (1)
Miscellaneous (14)	Genital tumor (5)	Glanulosa cell tumor (2)<br>Dysgerminoma (1)<br>Adenocarcinoma (1)<br>Seminoma (1)	Ovary (4)<br>Testis (1)
	Other endcrine tumor (4)	Pituitary tumor (2)<br>Pheochromocytoma (2)	Pituitary gland (2)<br>Adrenal gland (2)
	Odontogenic tumor (5)	Peripheral odontogenic fibroma (2)<br>(Fibromatous epulis)<br>Periferal odontogenic fibroma (2)<br>(Ossifying epulis)<br>Acanthomatous ameloblastoma (1)<br>(Acanthomatous epulis)	Oral cavity (5)
Total (191)			

Supplemental Table 1. Histopathological diagnosis and primary sites of tumours

The case numbers of each diagnosis and primary site are indicated in parentheses.

**Statistical methods**	**Figures**	**Softwares**
Kruskal-Wallis test	Figure 1b, c, d, e	GraphPad Prism 7
Chi-square Test	Figure 1f, Figure 4	GraphPad Prism 7
Mann-Whitney U test	Figure 2a, Figure 3a	GraphPad Prism 7
Steel Test	Figure 2b, c, Figure 3b, c	JMP Version 12, 64-bit
ROC curve analysis	Figure 5ab	GraphPad Prism 7
Cluster analysis, dendrogram, and heatmap illustration	Figure 2e, Figure 3e, Figure 4	JMP Version 12, 64-bit
Multivariate correlation analysis	Figure 7	JMP Version 12, 64-bit
Linear regression analysis	Figure 7	GraphPad Prism 7

Supplemental Table 2. Summary of statistical methods used in this study

Nine statistical methods were used in this study.

		**Disease**	**Sample type**	**Masured miRNA fraction**	**Separation method**	**Quantification**	**Evaluated clinical significance**	**Levels in Canine Tumors**	**References**
miR-214	Increased	Breast cancecr	Serum	Total circulating miRNAs	-	qRT-PCR	Diagnosis, LN metastasis	Increased	Schwarzenbach, H. *et al.*
		Gastric cancer	Plasma	Total circulating miRNAs	-	qRT-PCR	Diagnosis, Distant metastasis	-	Zhang, K.-C. C. *et al.*
		Myeloma	Serum	Total circulating miRNAs	-	qRT-PCR and Array	Diagnosis, Prognosis	-	Hao, M. *et al.*
		Osteosarcoma	Plasma	Total circulating miRNAs	-	qRT-PCR	Diagnosis, Prognosis	Increased	Allen-Rhoades, W. *et al.*
		Ovarian cancer	Serum	Exosome	MACS with anti-EpCAM	Array	Diagnosis	-	Taylor, D. D. *et al.*
	Decreased	Coronary artery disease	Plasma	Total circulating miRNAs	-	qRT-PCR	Signiture only	-	Lu, H. Q. *et al.*
miR-126	Increased	Allergic rhinitis and asthma	Plasma	Total circulating miRNAs	-	qRT-PCR	Signiture only	-	Panganiban, R. P. *et al.*
		Metastatic sporadic melanoma	Plasma	Exosome	ExoQuick solution	qRT-PCR and Array	Signiture only	Increased	Pfeffer, S. R. *et al.*
		Moyamoya disease	Serum	Total circulating miRNAs	-	qRT-PCR and Array	Signiture only	-	Dai, D. *et al.*
		Non-small cell lung carcinoma	Serum	Total circulating miRNAs	-	qRT-PCR	Diagnosis, Early diagnosis	-	Zhu, W. *et al.*
	Decreased	Atherosclerosis	Whole blood	Total blood miRNAs	-	qRT-PCR	Diagnosis	-	Jiang, Y. *et al.*
		Atrial fibrillation and heart failure	Serum	Total circulating miRNAs	-	qRT-PCR	Signiture only	-	Wei, X. J. *et al.*
		Type-2 diabetes mellitus	Serum	Total circulating miRNAs	-	qRT-PCR	Diagnosis, Treatment response	-	Liu, Y. et al. and Olivieri, F. *et al.*

Supplemental Table 3. Summary of previously reported human diseases with dysregulated levels of circulating miR-214 and -126

Human myeloma, osteosarcoma, breast, gastric, and ovarian cancers reportedly showed increased levels of circulating miR-214. The levels of circulating miR-126, in contrast, was increased in human diseases, such as allergic rhinitis and asthma, metastatic sporadic melanoma, Moyamoya disease, and non-small cell lung carcinoma, but was decreased in atherosclerosis, atrial fibrillation, heart failure, and type-2 diabetes mellitus. The term “Total blood miRNAs” refers to cellular miRNAs in white blood cells and cell-free circulating miRNAs in the plasma. The term “Total circulating miRNAs” from plasma or serum included protein-binding miRNAs and miRNAs contained in extracellular vesicles such as exosomes and microvesicles. MACS, magnetic-activated cell sorting; qRT-PCR, quantitative reverse transcription polymerase chain reaction.
